# 

**Published:** 1893-12-30

**Authors:** 


THE HOSPITAL.

PRICE TWOPENCE.

No. 379, Vol. XV.—Dec. 30th, 1893.

[Registered at the General Post Office as a Newspaper for transmission in the United Kingdom and Abroad.]

December 30th, 1[illegible]

WITH EXTRA NURSING SUPPLEMEN[illegible]

Per Annum, 8s. 6d., post free.

Monthly Parts, 6d.; post free, 8d.

Ditto per Annum, 8s., post free.

### THE HOSPITAL

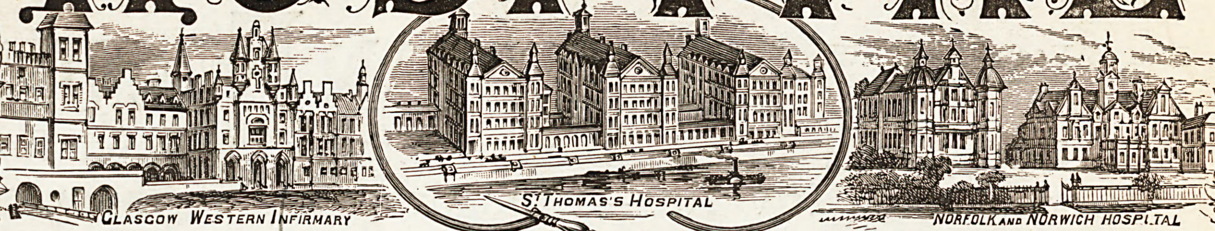

WITH EXTRA NURSING SUPPLEMENT.

No. 379. Vol. XV. Saturday, December 30th, 1893. [Twopence.

